# Dopamine agonist-associated fibrosis in prolactinomas: clinical challenges, impacts on the tumor microenvironment, and future directions

**DOI:** 10.3389/fendo.2026.1943425

**Published:** 2026-09-22

**Authors:** Chunxiao Qi, Lei Lei, Siyuan Lu, Xilong Wang, Peng Yu, Huanyu Zhu, Yong Wang, Yibao Wang, Shaowu Ou

**Affiliations:** 1 Department of Neurosurgery, the First Hospital of China Medical University, Shenyang, China; 2 Department of Geriatric Medicine, the Fourth People’s Hospital of Shenyang, Shenyang, China; 3 Department of Neurosurgery, Wafangdian Central Hospital, Wafangdian, China

**Keywords:** dopamine agonists, fibrosis, immune tolerance, prognosis, prolactinomas, tumor microenvironment

## Abstract

Prolactinomas account for approximately 30% to 50% of pituitary adenomas. Dopamine agonists (DAs) are the first-line treatment and effectively induce tumor regression while normalizing prolactin levels. However, DA therapy not only inhibits tumor growth but also is associated with fibrosis within the tumor. This fibrotic transformation remodels the tumor microenvironment (TME), altering cellular interactions, vascular structures, and extracellular matrix (ECM) composition. We hypothesize that this fibrotic response may not be a mere side effect; emerging, though largely indirect, evidence suggests it could potentially protect residual tumor cells by impeding drug penetration and facilitating immune evasion. Despite extensive clinical use of DAs, the mechanisms underlying DA-associated fibrosis and its implications for tumor behavior as well as patient outcomes remain poorly understood. This review integrates current knowledge regarding the pathophysiological processes leading to DA-associated fibrosis in prolactinomas, explores alterations in the TME, and assesses the clinical implications of these alterations. By integrating recent experimental and clinical studies, we aim to provide a comprehensive overview that elucidates the challenges posed by this fibrotic change in DA therapy and to propose actionable avenues for future research aimed at overcoming these obstacles.

## 1 Background

Prolactinomas (also referred to as prolactin pituitary neuroendocrine tumors (PitNETs)) represent the most common subtype of functioning pituitary adenomas, accounting for approximately 30% to 50% of clinically diagnosed cases (1, 2). These benign tumors arise from lactotroph cells in the anterior lobe of the pituitary and are characterized by excessive prolactin (PRL) secretion. Hyperprolactinemia manifests clinically as galactorrhea, menstrual irregularities in women, hypogonadism, infertility, reduced libido, and, in larger tumors, symptoms related to mass effects such as headache and visual field deficits (3–5). Dopamine agonists (DAs), including cabergoline and bromocriptine, constitute the first-line treatment for prolactinomas (6, 7). By activating dopamine D2 receptors (D2R) on lactotrophs, DAs effectively normalize serum prolactin levels and promote tumor shrinkage in most patients (1, 8). Beyond hormonal regulation, D2R activation modulates cell cycle progression and induces apoptosis in tumor cells, which underlies the significant tumor reduction observed in clinical practice (9, 10).

Nevertheless, some patients show resistance or intolerance to DAs, necessitating alternative interventions such as surgical resection or radiotherapy (7, 11). The mechanisms of DA resistance are complex and not fully elucidated, involving alterations in D2R expression and signaling, tumor fibrosis, microRNA dysregulation, and receptor heteromerization, which collectively impair dopaminergic signaling and contribute to therapeutic failure (12). Despite the overall efficacy of DAs, prolonged use of DAs may inadvertently be associated with fibrosis within residual tumors (13, 14). Over recent decades, clinical and histopathological studies have documented tumor fibrosis in prolactinomas during or following DA therapy (13–17). Bromocriptine has long been implicated in promoting fibrosis, whereas the role of cabergoline remains debated; however, recent evidence supports its potential contribution to fibrotic transformation (13, 18, 19). This phenomenon is likely attributable to differential apoptotic mechanisms elicited by these agents, as well as the limited number of reported cases (20). Fibrotic tumors exhibit increased collagen deposition, reduced responsiveness to medical therapy, and greater surgical challenges, resulting in prolonged operative times and increased complication rates (14, 16, 21). Importantly, fibrosis extends beyond structural alteration, actively remodeling the tumor microenvironment (TME) by activating stromal cells and modifying the extracellular matrix (ECM) (13, 18).

The TME critically influences prolactinoma behavior and therapeutic response (22, 23). Fibrosis within the TME alters tumor cell interactions, ECM composition, and mechanical properties, affecting drug penetration, immune cell infiltration, and local inflammatory responses (24, 25). These alterations may further modulate macrophage polarization and T cell infiltration, facilitating immune evasion and promoting tumor progression (26, 27). These findings give rise to the hypothesis—though not yet directly demonstrated in prolactinomas—that DA-associated fibrosis may not be solely a treatment side effect but could represent an adaptive tumor response that establishes a protective fibrotic niche. It has been proposed that such a niche might impede drug delivery, alter immune surveillance, and increase tissue rigidity, thereby contributing to treatment resistance (28, 29). An alternative, yet equally plausible, interpretation posits that fibrosis results from tumor involution, apoptosis, and the ensuing tissue remodeling, rather than an adaptive resistance mechanism. However, this remains speculative and necessitates experimental validation in prolactinoma-specific models. Consequently, DA-associated fibrosis poses a significant clinical challenge, warranting reconsideration of risk-benefit assessments, particularly regarding surgical timing. Some studies advocate early surgical intervention in selected patients to circumvent complications from fibrotic transformation (14, 16, 30), although this approach must be balanced against the high efficacy and non-invasiveness of DA therapy for the majority (31, 32). The development of robust biomarkers to predict fibrosis risk is urgently needed. Circulating microRNAs, such as miR-20a-5p, are being explored to predict DA responsiveness and monitor therapeutic effects, facilitating individualized treatment strategies (33).

In this review, “DA-associated fibrosis” is defined as increased collagen deposition and stromal remodeling observed in prolactinomas following DA therapy. This term comprises several distinct but potentially interrelated phenomena: (i) perivascular fibrosis, (ii) diffuse stromal collagen deposition, (iii) radiological changes indicating altered tumor consistency, and (iv) intraoperative tumor firmness. These categories are not invariably concordant, and their clinical significance may vary. We synthesize evidence relevant to the hypothesis that fibrotic remodeling establishes a protective niche within the prolactinoma TME, shielding tumor cells from both pharmacological and immunological attack. We summarize the clinical and pathological data supporting this phenomenon, analyze the structural and functional aspects of the fibrotic barrier, and examine underlying molecular mechanisms. Finally, we discuss emerging strategies to prevent or mitigate fibrotic change to optimize patient outcomes. A critical caveat of this review is that the relationship between DA-associated fibrosis and therapeutic resistance is likely bidirectional. Most histological evidence is derived from surgically treated patients with DA-resistant or difficult-to-control disease. Therefore, it remains unclear whether DA exposure promotes fibrosis that subsequently contributes to resistance, whether resistant tumors are subjected to prolonged DA exposure and consequently develop more fibrosis, or whether aggressive tumor biology independently contributes to both resistance and fibrosis. Reverse causation and confounding by factors such as tumor size, invasiveness, DA type, dose, and treatment duration must be considered when interpreting the available data.

## 2 Clinical implications and challenges of DA treatment in prolactinoma patients

Bromocriptine and cabergoline are employed as the first-line treatments for prolactinomas, effectively decreasing PRL levels and tumor size. Cabergoline, a more recent ergot derivative, demonstrates higher receptor affinity and a longer half-life than bromocriptine. These pharmacokinetic properties enhance patient adherence and improve both biochemical and radiographic outcomes. In clinical observations, PRL levels normalize following cabergoline treatment in 90% of patients with microprolactinomas and 83% of those with macroprolactinomas. Furthermore, adenoma shrinkage occurs in 71% of cases for both microadenomas and macroadenomas (8, 34). In contrast, bromocriptine generally demonstrates lower efficacy, particularly in reducing macroadenoma size, and is frequently associated with adverse effects, including nausea, headaches, and dizziness, which may lead some patients to discontinue therapy (2, 35).

Despite the overall efficacy of DAs, several patients exhibit resistance or intolerance to DA treatment. Resistance, defined as the inability to normalize PRL levels or achieve a tumor size reduction of at least 50% despite adequate dosing and sufficient treatment duration, occurs in approximately 10%-30% of cases, depending on the specific agent and patient demographic (1, 36, 37). Long-term DA therapy is generally well tolerated; however, side effects such as gastrointestinal disturbances, valvulopathy, and impulse control disorders (ICDs) can occur, though they are rare in prolactinoma patients (38, 39). Gastrointestinal symptoms are the most common, affecting up to one-third of patients (36). ICDs, such as compulsive shopping and hypersexuality, are less frequent but clinically significant, with variable prevalence (39). Moreover, secondary resistance or disease relapse after an initial response often necessitates surgical intervention (2).

Drug-related adverse events and tumor resistance are primary contributors to DA treatment failure, but DA-associated tumor fibrosis significantly affects the efficacy of subsequent treatment. The relationship between fibrosis and DA resistance is complex and potentially bidirectional (30, 40, 41). Whether fibrosis precedes resistance, results from prolonged therapy in refractory cases, or is simply correlated with treatment exposure remains unresolved. Reverse causation and confounding by tumor size, invasiveness, DA type, dose, and treatment duration must be carefully accounted for. For patients who are resistant or intolerant to DAs, surgical intervention remains a key alternative, although optimal timing and indications remain debated (7). Some retrospective studies have reported an association between presurgical DA administration and increased tumor fibrosis (14, 16, 17); in some cohorts, fibrosis was associated with greater operative difficulty and lower remission rates (14). However, these studies are limited by retrospective design, selection bias, variability in DA type, dose, and duration, lack of standardized fibrosis grading, and inter-surgeon variability. The independent predictive value of fibrosis for surgical outcomes remains uncertain. Some researchers argue that tumor fibrosis is becoming less of a concern due to the application of modern DAs, such as cabergoline, and advances in endoscopic surgical techniques (32). However, this view may be excessively optimistic, particularly for macroprolactinomas. Fibrosis alters the surgical plane by hiding the pseudocapsule, thereby complicating the pseudocapsule-based resection—a prerequisite for biochemical remission in macroadenomas (42, 43). This complexity increases the risk of cerebrospinal fluid leakage and other complications. The available literature is heterogeneous, and to date, no randomized controlled trials (RCTs) have investigated this issue and most evidence regarding DA-associated fibrosis derives from retrospective surgical series, which are susceptible to selection bias and lack standardized histopathological grading.

Consequently, there is an urgent need for objective preoperative methods to assess tumor consistency in DA-exposed prolactinoma patients. Conventional imaging techniques, such as T2-weighted magnetic resonance imaging (MRI), have proven inadequate for accurately evaluating tumor consistency due to poor correlation with histological fibrosis (44). Magnetic resonance elastography (MRE) is currently under investigation as a tool to assess tumor stiffness in pituitary adenomas (45, 46). MRE has the potential to provide objective preoperative assessment of fibrosis in PRL-secreting adenomas, thereby facilitating the determination of optimal surgical timing. Achieving this requires comprehensive clinical data collection and detailed analysis of molecular mechanisms underlying pathological changes. Future strategies will likely involve integrating advanced imaging modalities with molecular markers to predict individual fibrosis risk, guiding clinical decisions on prolonged DA therapy versus timely surgical intervention. Such approaches demand a multidisciplinary effort to optimize long-term outcomes for patients with prolactinomas.

## 3 Evidence of DA-associated fibrosis in treating prolactinomas

It is important to recognize that most historical evidence pertains to bromocriptine, whereas evidence regarding intratumoral fibrosis associated with cabergoline, although emerging, remains more limited and inconsistent. The following section therefore distinguishes between these agents where sufficient data exist (**Table 1**). Tumor fibrosis associated with DA therapy is a major factor contributing to the increased firmness of prolactinomas. Landolt et al. first reported an association between DA treatment and fibrosis, observing that tumor shrinkage coincided with collagen deposition in enlarged extracellular and perivascular spaces (17). Subsequent studies by Esiri and Takahashi confirmed a significant correlation between prolonged DA therapy and increased tumor fibrosis (47, 48). Histological analyses of prolactinoma specimens from DA-treated patients consistently demonstrate substantial collagen fiber accumulation and stromal proliferation, assessed using advanced methods such as Masson’s trichrome staining combined with artificial intelligence-based imaging software (14). It is important to note that most investigations to date have focused on bromocriptine, whereas evidence regarding cabergoline remains limited and warrants further evaluation (14, 17, 19, 47–49). A case series reported markedly higher fibrosis levels in cabergoline-pretreated patients (50%-70% by Masson’s trichrome staining) compared with approximately 15% in untreated tumors (13). Nevertheless, other studies indicate that standard cumulative doses of cabergoline pose minimal systemic fibrotic risk (51, 52), suggesting that fibrosis outcomes are context-dependent and influenced by treatment duration, dosage, and individual susceptibility, although the limited number of reported cases precludes definitive conclusions. Rare but clinically significant complications, such as cardiac valvular and pulmonary fibrosis, underscore the importance of careful monitoring (52, 53). Some clinical studies have correlated DA-associated tumor fibrosis with increased intraoperative tumor firmness, longer surgical time, and greater blood loss (14, 16). However, these associations may be confounded by tumor size, invasiveness, DA type, dose, and treatment duration. Furthermore, reverse causation is possible, as larger or more aggressive tumors may require longer or higher-dose DA treatment and are potentially more fibrotic at baseline.

**Table 1:** Evidence from previous studies indicates the association between presurgical DA treatment and prolactinoma fibrosis.

Author and year (reference)	Sample size	DA types	Methods for fibrosis assessment	Pretreatment duration (total or daily dose of DA)	Evidence type	Tumor fibrosis
Landolt et al, 1984 (17)	21	BC	Electron microscope	3 months-2 years(2.5-7.5 mg/day, TD: 450-5500mg)	Patient samples	Yes
Esiri et al, 1986 (47)	7	BC	Light microscope, Gordon and Sweet’s method	3 weeks-9 months (NA)	Patient samples	Yes
Takahashi et al, 1986 (48)	15	BC	Electron mircoscope	30–880 days (5-30mg/day)	Patient samples	Yes
Bevan et al, 1987 (49)	15	BC	Operative observation	3–48 weeks(10-40mg/day)	Patient samples	Yes
Menucci et al, 2011 (19)	21	BC/CAB	Operative observation	BC:30–550 days (TD: 75-1375mg); CAB:42–989 days (TD: 6-70mg)	Patient samples	Yes
Chen et al, 2024 (14)	158	BC/CAB	Masson trichrome staining	BC: 3–24 months (TD: 450-3150mg); CAB: 3–24 months (TD: 30-384mg)	Patient samples	Yes
Pecorari et al, 2024 (13)	3	CAB	Masson trichrome staining and AI imaging software	1–6 months (0.5mg, twice weekly)	Patient samples	Yes
Zhang et al, 2024 (18)	3	CAB	Single-cell RNA sequencing (scRNA-seq)	NA (2mg, weekly)	Patient samples	A higher stromal cell population was found
Mamelak et al, 2026 (50)	37	BC/CAB	Masson trichrome staining	16–7830 days (TD: 5.4-6711mg)	Patient samples	No significance

DA, Dopamine agonist; BC, Bromocriptine; CAB, Cabergoline; NA, Not acquired; AI, Artificial intelligence; TD, Total dose.

Experimental studies using animal models and *in vitro* systems have begun to clarify the mechanisms underlying DA-associated fibrosis. The effects of DA appear to be tissue-specific and receptor-dependent. In cardiac tissue, dopamine D3 receptor (D3R) activation by agonists such as pramipexole promotes fibroblast migration and proliferation, key processes in fibrogenesis (54). In contrast, pulmonary fibrosis models demonstrate that D1R and D2R agonists attenuate fibroblast activation and ECM deposition through inhibition of TGF-β1/Smad2 signaling (55). These findings suggest that fibrotic responses are modulated by the specific dopamine receptor subtypes expressed in each tissue and the local microenvironment. In prolactinomas, D2R predominance may drive a pro-fibrotic phenotype, although this hypothesis requires direct validation. Employing advanced techniques such as single-cell RNA sequencing and specialized animal models will be essential to transition from correlative observations to mechanistic insights (18). Such mechanistic understanding is expected to inform clinical strategies that optimize treatment outcomes while minimizing fibrotic complications.

## 4 Mechanisms underlying DA-associated fibrosis in prolactinomas

The development of fibrosis in prolactinomas following DA treatment is orchestrated by a complex interplay of cytokines and signaling molecules, and is potentially associated with therapeutic resistance. Notably, DA resistance is more strongly associated with tumor biology, D2 receptor expression and signaling, invasion, and molecular alterations than with fibrosis itself (36, 37, 56). The role of fibrosis in DA resistance remains to be fully elucidated. One proposed underlying mechanism for DA-associated fibrosis in prolactinomas is that DA treatment induces apoptosis in prolactinoma cells, leading to cellular shrinkage and passive enlargement of extracellular and perivascular spaces, which are subsequently filled with deposited collagen (17, 20). However, this passive mechanism does not fully explain the active fibroblast proliferation and ECM remodeling observed in certain cases. Although the exact molecular mechanisms underlying this process have not been fully elucidated, transforming growth factor-beta (TGF-β) and connective tissue growth factor (CTGF) have emerged as central mediators of this pathway (**Figure 1**).

**Figure 1:**
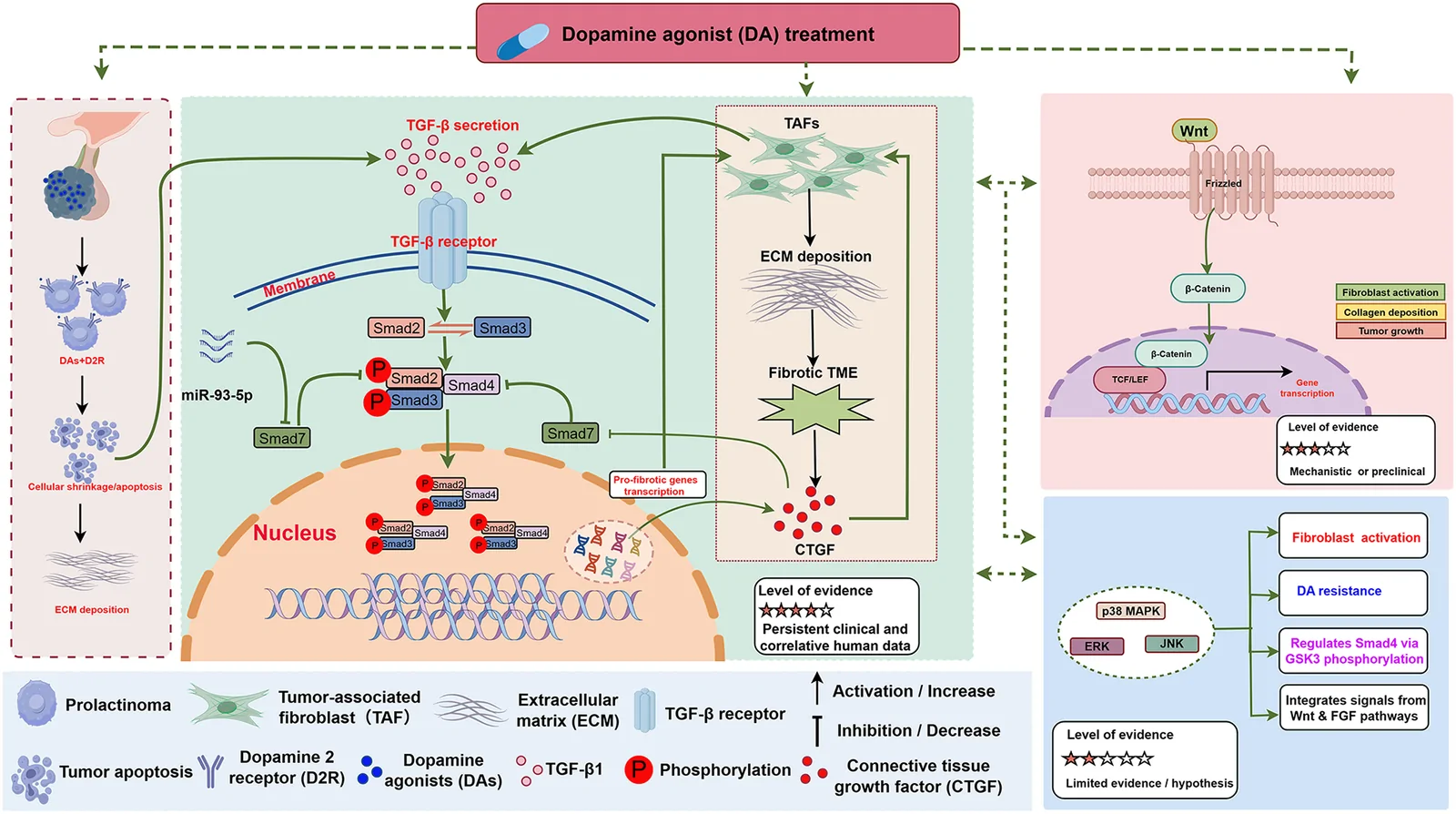
Potential mechanisms underlying DA-associated fibrosis in prolactinomas, placing particular emphasis on the central role of TGF-β signaling, including levels of evidence. Evidence levels are indicated by stars: four stars, persistent clinical and human correlative data; three stars, mechanistic or clinical data; two stars, limited data or hypotheses.

TGF-β, a multifunctional cytokine with three isoforms (TGF-β1, TGF-β2, and TGF-β3), is well-established as a master regulator of fibrosis by promoting ECM deposition and activating fibroblasts into myofibroblasts (57). The role of TGF-β and receptors in tumor fibrosis, including associated downstream signaling pathways, is extensively documented (22, 58, 59). A previous study indicated that TGF-β1 expression in fibrotic prolactinoma was approximately 3.3-fold higher than in nonfibrotic tumors (60). DA treatment, acting via the D2R on lactotrophs, upregulates TGF-β1 expression and secretion both *in vivo* and *in vitro*, while concurrently inhibiting lactotroph proliferation (57, 61). Consistent with a pathogenic role in tumor progression, components of the TGF-β1/Smad3 (mothers against decapentaplegic homolog 3) pathway are elevated in DA-resistant, highly fibrotic tumors compared to DA-sensitive tumor specimens (62). Binding of TGF-β and its receptor activates the canonical Smad signaling cascade. *In vitro*, co-culture of rat prolactinoma MMQ cells with TGF-β1-treated HS27 fibroblasts reduced bromocriptine sensitivity, while TGF-β1 stimulation increased collagen expression in HS27 cells (62). In fibrotic prolactinomas, increased phosphorylation of Smad2 and Smad3 is observed, followed by complex formation with Smad4 and their subsequent nuclear translocation, which promotes the transcription of pro-fibrotic genes and upregulates type I and type III collagen expression (63). This process is tightly regulated by multiple molecules or pathways; for example, Smad7 functions as a negative regulator by interacting with activated TGF-β receptor 1 (TβRI), thereby inhibiting Smad2 phosphorylation and TGF-β1/Smad3-dependent fibrosis (64). Notably, miR-93-5p downregulates Smad7 expression, ultimately promoting TGF-β1/Smad3-mediated fibrogenesis (57, 60, 62).

CTGF, a key downstream effector of TGF-β signaling, amplifies fibrogenesis by stimulating collagen synthesis and fibroblast proliferation (65, 66). This TGF-β/CTGF axis may contribute to the formation of a dense fibrotic stroma that characterizes DA-resistant prolactinomas and other refractory pituitary tumors. Tumor-associated fibroblasts (TAFs) secrete TGF-β and CTGF, perpetuating fibrosis and inflammation (22, 58). This persistent activation recruits and activates TAFs, which secrete additional TGF-β and CTGF, thereby creating a self-reinforcing cycle of fibrosis that supports tumor growth and therapeutic resistance (67, 68). In addition, TGF-β-induced endogenous CTGF promotes transcriptional repression of Smad7, thereby blocking the inhibitory effect of Smad7, resulting in persistent activation of TGF-β signaling (69). Given its central role, CTGF represents a potential biomarker of disease progression and a therapeutic target in DA-resistant prolactinomas. Further studies are warranted to elucidate the mechanisms by which CTGF mediates fibrotic remodeling.

The role of TGF-β1 in prolactinomas, however, presents a paradox. Although its pro-fibrotic function in treated tumors is well-established, certain studies indicate that the levels of TGF-β1 and Smad2/3 are actually reduced in untreated prolactinomas compared to normal anterior pituitary tissue, implying its potential tumor-suppressive role during early tumorigenesis (57, 70, 71). This duality presents a considerable challenge for therapeutic targeting. A major obstacle in resolving this paradox is the significant lack of dynamic human tissue data. Surgically excised, fibrotic tumors typically represent the DA-resistant end, whereas DA-sensitive tumors, which regress without substantial fibrosis, are infrequently biopsied. This selection bias significantly limits our understanding of the temporal dynamics and critical threshold of TGF-β signaling that leads to a fibrotic pathway in tumors. Consequently, the development of non-invasive biomarkers and advanced disease models are an urgent priority.

Beyond the TGF-β axis, other signaling pathways may synergistically contribute to DA-associated fibrosis (**Figure 1**). The Wnt/β-catenin pathway is known to activate fibroblasts and increase collagen deposition, which is often elevated in DA-resistant prolactinomas, potentially boosting tumor growth and fibrosis (22). This pathway interacts with TGF-β signaling, as evidenced by the requirement of canonical Wnt signaling for TGF-β-mediated fibrosis, indicating a synergistic relationship between these pathways in fibrotic disease progression (72). The interplay between these pathways is further complicated by the involvement of mitogen-activated protein kinase (MAPK) signaling. The MAPK pathway, which includes extracellular signal-regulated kinase (ERK), the c-Jun N-terminal kinase (JNK), and p38 MAPK, is activated in response to TGF-β signaling and contributes to the fibrotic response by modulating fibroblast activity and ECM deposition (73). The MAPK pathway also contributes to the resistance of prolactinomas to DAs. Immunofluorescence analysis showed that PRL and MAPK14 (p38α MAPK) expression were colocalized and increased in human prolactinoma specimens (74). In an *in vitro* study, knockdown of MAPK11 (p38β MAPK) and MAPK14 in DA-resistant GH3 cells reversed the resistance of GH3 cells to bromocriptine (75). Additionally, the MAPK pathway can influence the activity of the Wnt pathway, as demonstrated by the regulation of Smad4, a key mediator of TGF-β signaling, through MAPK and glycogen synthase kinase 3 (GSK3) phosphorylations, which integrate signals from Wnt and fibroblast growth factor (FGF) pathways (76). This integration underscores the complexity of signaling networks in fibrosis, where multiple pathways converge to regulate cellular responses. Moreover, dopamine receptors are expressed on fibroblasts themselves, and their activation or inhibition can directly alter fibroblast behaviors (54, 55). Whether direct D2R signaling in prolactinoma fibroblasts contributes to fibrosis remains a hypothesis that requires validation through further investigation. Furthermore, alterations in the subpopulations of fibroblasts within prolactinomas following DA treatment remain inadequately understood. The implementation of single-cell sequencing technology has the potential to offer novel insights into addressing this issue. Collectively, these findings raise an intriguing hypothesis: DA resistance might partly result from a stromal reprogramming, in which DAs, acting through fibroblast D2R, may directly instigate a pro-fibrotic TME. Investigating this hypothesis requires advanced co-culture systems or fibroblast-specific D2R knockout models. Therefore, given these considerations, the potential impact of direct dopamine receptor signaling on prolactinoma fibroblasts warrants dedicated investigation.

These studies collectively show that dopamine receptor signaling engages in complex interactions with several fibrosis-related pathways. Current evidence most consistently implicates the TGF-β/Smad axis in DA-associated fibrosis, but definitive evidence establishing it as the primary driver in human prolactinomas remains lacking. Other mechanisms—including DA-induced cell shrinkage and apoptosis with passive collagen filling of enlarged extracellular and perivascular spaces, direct D2R-mediated fibroblast activation, and cross-talk with Wnt/β-catenin and MAPK pathways—may operate either in parallel or sequentially, supported by varying degrees of evidence. Future research should aim to elucidate the specific roles of each pathway and their crosstalk within the prolactinoma TME. Ultimately, deconstructing the fibrotic niche may require a multi-pronged strategy. Targeting the TGF-β/CTGF axis (the potential central pro-fibrotic driver) alongside specific dopamine receptor signaling (the potential stromal trigger) could provide a novel therapeutic paradigm to prevent or reverse fibrosis, thereby improving pharmacological efficacy and surgical outcomes.

## 5 TME changes in fibrotic prolactinomas

### 5.1 The effects of fibrosis on prolactinoma cell proliferation and apoptosis

In addition to its structural function, the rigid ECM in fibrotic prolactinomas may serve as a dynamic signaling platform that modulates tumor cell fate, although this hypothesis is primarily extrapolated from other tumor types and has yet to be empirically validated in prolactinomas. Fibrotic remodeling increases the ECM stiffness and modulates mechanical signaling pathways that regulate tumor cell fate (68, 77, 78). Increased ECM rigidity affects mechanotransduction by modulating integrin-mediated adhesion and downstream signaling cascades, including focal adhesion kinase (FAK) and MAPK pathways. In DA-resistant prolactinomas, activation of the FAK pathway has been observed (36). ADAM metallopeptidase domain 22 (ADAM22), a disintegrin and metalloproteinase, is upregulated in pituitary adenomas, interacts with integrin β1 to activate FAK-mediated phosphatidylinositol-4,5-bisphosphate 3-kinase (PI3K) and ERK signaling pathways, promoting tumor cell proliferation and invasion (79). This suggests that fibrotic changes in the ECM can enhance integrin signaling, thereby tipping the balance towards increased proliferation and survival of tumor cells (77, 80).

The pro-survival impact of this fibrosis-driven mechanical signaling is profoundly amplified by its convergence with intrinsic defects in the dopaminergic pro-apoptotic signaling. While DAs normally induce apoptosis by inhibiting protein kinase B (AKT) via a β-arrestin 2-dependent mechanism (81, 82), resistant cells exhibit altered signaling, including upregulation of pro-survival p38 MAPK isoforms (74, 75). The stiff fibrotic ECM creates a synergistic loop: its sustained activation of PI3K/AKT and ERK pathways crosstalks with and overrides the impaired pro-apoptotic signals from DAs, potentially shielding tumor cells from programmed death.

Collectively, these intertwined signaling cascades culminate in a state of profound therapeutic resistance. Specifically, increased ECM rigidity promotes drug resistance and enhances invasive potential by activating integrin-FAK signaling, as well as downstream effectors such as ERK and PI3K/AKT pathways (36, 79). The focal adhesion (FA) pathway has been identified as upregulated in DA-resistant prolactinomas, and its inhibition by compounds like genistein suppresses tumor cell proliferation and induces apoptosis, suggesting the role of ECM-related signaling in therapeutic resistance (36). While current evidence is largely correlative, it is consistent with the hypothesis that fibrosis may not be simply an inert scar tissue, but could actively contribute to a pro-survival niche. Nevertheless, a causal relationship has not yet been established. This may partly explain the observed association between fibrosis and therapy resistance, positioning fibrosis as a potentially active driver of tumor adaptation and treatment failure. However, current evidence remains insufficient to determine whether fibrosis is a causal factor or downstream effect of DA resistance.

### 5.2 Activation of TAFs in fibrotic prolactinomas

In the context of the dynamic fibrotic change observed in DA-treated prolactinomas, TAFs are hypothesized to contribute to stromal remodeling. TAFs have been implicated as contributors to tumor progression and fibrosis in various cancer types, originating from various sources, including resident fibroblasts, mesenchymal stem cells, tumor cells, endothelial cells and transdifferentiation processes (21, 64). These cells exhibit distinct phenotypes and functions that are dependent on the specific TME (83, 84). Although TAFs have a well-documented role in carcinomas and malignant progression (85), their function in pituitary adenomas remains poorly understood and these models should not be directly extrapolated to prolactinomas uncritically. In pituitary adenomas, the cellular origins and functional states of TAFs remain to be well-defined, and it remains unclear whether they are analogous to carcinoma-associated fibroblasts (CAFs) described in epithelial cancers (85). Current evidence indicates a correlation between the degree of TAF activation and tumor behaviors (85, 86).

Notably, TAFs in invasive pituitary adenomas demonstrate elevated expression levels of α-smooth muscle actin (α-SMA) and vascular endothelial growth factor (VEGF) (87). DAs may indirectly facilitate fibrosis in prolactinomas by inducing tumor cells to secrete cytokines and growth factors (e.g., TGF-β, FGFs), as well as other profibrotic mediators that activate TAFs (**Figure 2**). TAFs have been reported to produce TGF-β, FGF2, and additional cytokines or growth factors that are implicated in drug resistance and heightened inflammation in somatotroph and lactotroph tumors (22). In other tumors, factors secreted by TAFs serve as fundamental components in the construction of the fibrotic microenvironment, and TAFs have been shown to facilitate ECM deposition and cross-linking, potentially enhancing the physical barrier through increased stiffness, which impedes drug penetration (88, 89). It has also been proposed that TAF-derived factors may modulate immune cell infiltration and foster an immunosuppressive milieu (85, 90–92). Whether these functions persist in DA-treated prolactinomas remains to be determined. In other tumors, the secretory profile of TAFs encompasses ECM components such as collagen and fibronectin, alongside ECM-degrading enzymes (93–96). This combination plays a pivotal role in regulating the equilibrium between ECM deposition and degradation, which is essential in the pathogenesis of fibrosis (93, 94). Furthermore, TAFs engage in reciprocal communication with tumor cells and other stromal elements through soluble factors and direct cell-cell interactions, thereby influencing tumor cell proliferation, invasion, and resistance to therapeutic interventions (97)(**Figure 2**). These interactions can enhance tumor stiffness and fibrosis, potentially affecting treatment efficacy and tumor dynamics. Elucidating the mechanisms underlying TAF activation by DA-associated tumor cell secretion will be important for formulating strategies to mitigate fibrosis and enhance therapeutic outcomes in prolactinoma patients (22). For example, interferon-γ (IFN-γ) has been reported to inhibit the remodeling of TAF from a myofibroblastic phenotype to an inflammatory/antigen-presenting state (98). Additionally, TAF-derived cytokines may increase pituitary adenoma aggressiveness, alter angiogenesis and induce epithelial-to-mesenchymal transition (EMT) changes (85). Pasireotide has demonstrated efficacy in suppressing TAF activity, leading to a reduction in TAF-derived cytokine levels (e.g., IL-6 and CCL2) in pituitary adenoma models (85). However, these findings do not specifically investigate DA-associated fibrosis in prolactinomas, and their translational applicability to this context has yet to be determined. This underscores the importance of first ascertaining whether distinct fibroblast subpopulations exist in DA-treated prolactinomas and whether these potential subpopulations are associated with fibrosis or therapeutic resistance. Spatial multi-omics approaches offer a promising avenue to address this inquiry; however, a validated “DA-resistant TAF” phenotype has not yet been established. Subsequently, it may be valuable to explore targeted strategies, potentially by disrupting specific feedback loops such as TGF-β/CTGF that sustain their pathological state, rather than depending on broad-spectrum reprogramming agents.

**Figure 2:**
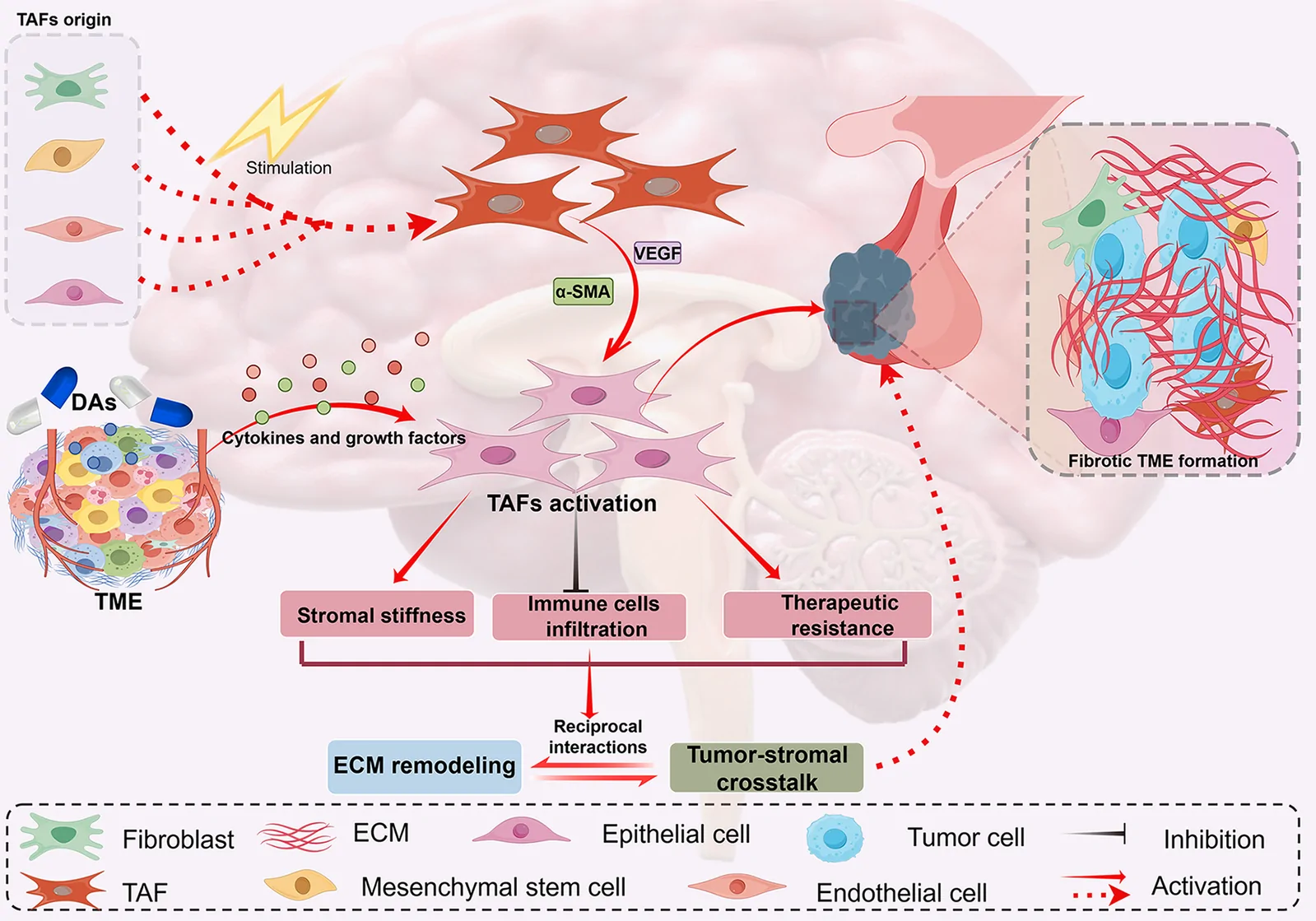
Potential mechanisms driving the activation of tumor-associated fibroblasts (TAFs) within the prolactinoma microenvironment during dopamine agonist (DA) treatment.

### 5.3 The immune microenvironment of fibrotic prolactinomas: a significant knowledge gap

Direct evidence characterizing the immune microenvironment of fibrotic prolactinomas is limited. The concepts discussed in this section are therefore largely derived from other pituitary adenomas or non-pituitary tumors and should be regarded as hypotheses rather than established pathological findings. Based on extrapolation from other tumor types, we hypothesize that fibrosis in prolactinomas may contribute to establishing an immunosuppressive niche through multiple, interconnected mechanisms: (i) the ECM forms a physical barrier that impedes immune cell infiltration; (ii) dysregulated secretion of factors such as TGF-β and PRL generates a biochemically suppressive landscape; and (iii) prolonged DA therapy may exert direct immunomodulatory effects (**Figure 3**). This hypothesis builds upon, yet is distinct from, the established immunological framework for pituitary adenomas.

**Figure 3:**
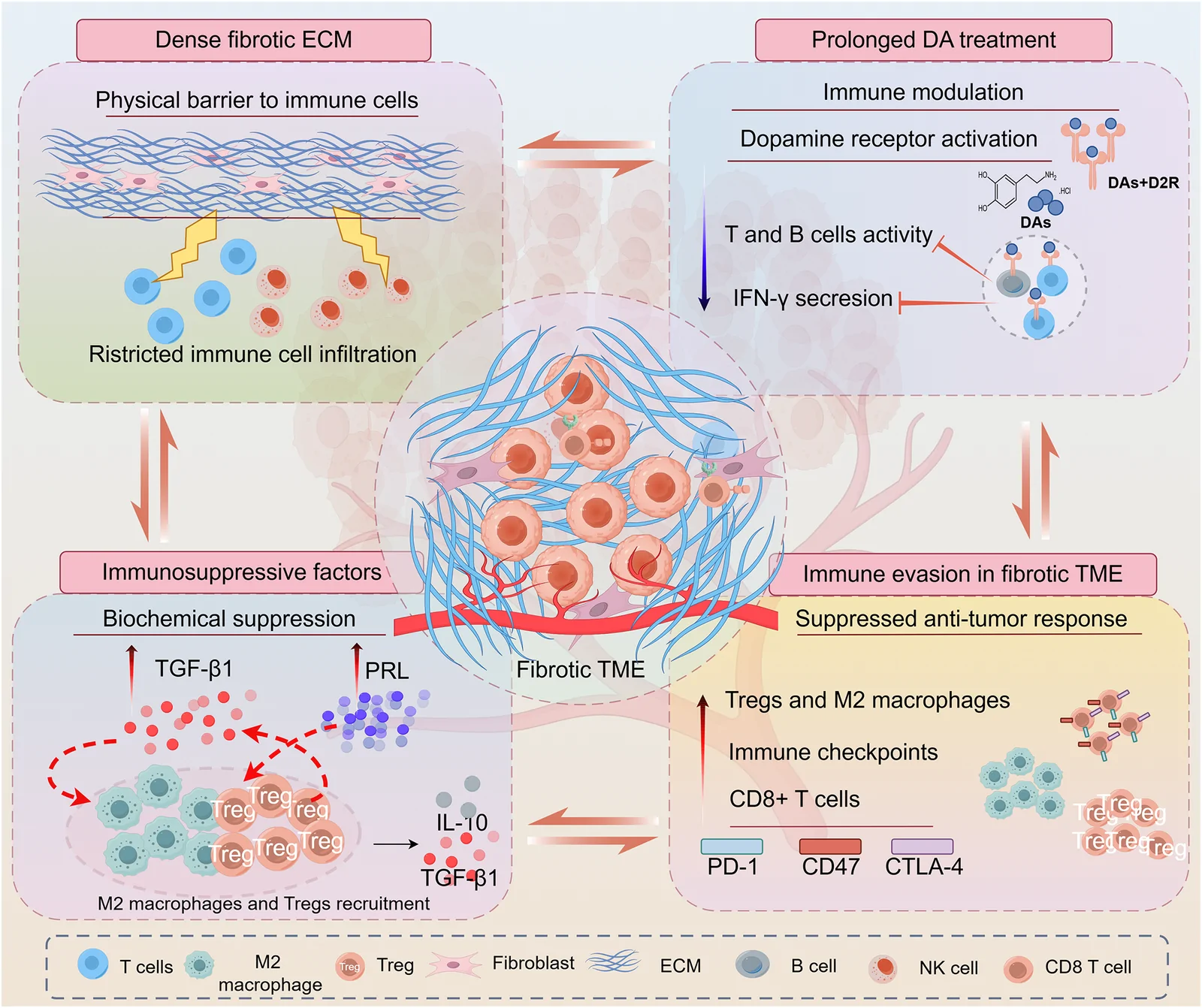
Potential alterations of the tumor immune microenvironment in the context of fibrosis associated with dopamine agonist (DA) treatment in prolactinomas.

Heterogeneity among pituitary-specific transcription factor-1 (PIT1) lineage adenomas (lactotroph, somatotroph, and thyrotroph) is well-documented (99–102). These adenomas commonly downregulate major histocompatibility complex class I (MHC-I) molecules (human leukocyte antigen-A (HLA-A), HLA-B, and HLA-C) and activate inflammatory and EMT pathways (98). Non-functioning pituitary adenomas similarly demonstrate attenuated local immune responses (103). Collectively, these findings indicate an intrinsic potential for immune suppression within the pituitary adenoma microenvironment. In fibrotic prolactinomas, we propose that fibrosis may reinforce this immunosuppressive state (**Figure 3**).

Treatment-naive prolactinomas exhibit a lymphoid-predominant immune profile (104, 105), unlike the myeloid-rich microenvironment of gliomas (106, 107). This disparity may stem from tumor invasiveness or blood-brain barrier (BBB) mechanisms within the TME (108, 109). Sex-specific patterns also exist: males show enrichment in resting dendritic cells and naive CD4+ T cells, associated with immune checkpoints such as CD47 molecule (CD47) and CEA cell adhesion molecule 1 (CEACAM1) (110). A central question remains: does fibrosis, often following DA therapy, amplify existing immunosuppression or induce a distinct cellular state?

Available data on prolactinomas are limited and often derived from small patient cohorts or populations with mixed pituitary tumors. For example, evidence from DA-sensitive prolactinomas indicates an increase in CD8+ T cells expressing cytotoxic granule proteins, including perforin and granzymes (granzyme B (GZMB) and granulysin (GNLY)), alongside inflammatory cytokines such as C-C motif chemokine ligand 5 (CCL5), following cabergoline treatment (18). Conversely, fibrotic prolactinomas likely represent a unique immunological state. Fibrosis creates a dense ECM that physically restricts the penetration of cytotoxic T lymphocytes (CTLs) and natural killer (NK) cells (25). Biochemically, TGF-β1 sustains fibrotic activity while inhibiting effector T cells and recruiting M2-polarized macrophages and regulatory T cells (Tregs) (111). In fibrotic tumors, Tregs and M2 macrophages have been reported to be enriched and exert potent immune suppression through anti-inflammatory cytokine secretion and immune checkpoint expression (112, 113). However, whether similar enrichment occurs in fibrotic prolactinomas specifically following DA therapy remains unclear*. In vitro*, conditioned medium from MMQ cells induces M2 polarization in tumor-associated macrophages (TAMs), facilitating tumor invasion via C-C motif chemokine ligand 17 (CCL17) secretion (114). These observations are primarily correlational and fail to establish a causal link between fibrosis and immune suppression. They underscore the necessity of prospective studies that directly compare immune profiles between fibrotic and non-fibrotic prolactinomas.

Persistent hyperprolactinemia often occurs in fibrotic prolactinomas. PRL levels correlate positively with the infiltration of CD4+ T cells, CD8+ T cells, γδT cells, and B cells in prolactinomas. Nevertheless, the functionality of these immune cells is often restricted by fibrotic septa, which reinforce an immune-suppressive environment and upregulate immune checkpoints, including programmed cell death 1 (PDCD1), CD38, and CD274 (programmed death-ligand 1, PD-L1) (115). Spatial transcriptomics combined with multiplexed fluorescent immunohistochemistry can enhance the precise localization of immune cells. Moreover, elevated expression of cytotoxic T-lymphocyte associated protein 4 (CTLA4), along with its mRNA level co-expression with the PRL protein in prolactinomas, further reflects immunosuppressive conditions (116). The immunomodulatory effects of PRL are dose-dependent and context-specific: low levels promote pro-inflammatory responses, whereas high or persistent levels suppress effector T-cell function, particularly under prolonged hyperprolactinemia typical of DA-resistant fibrotic tumors (117–119).

DAs may exert immunomodulatory effects via dopaminergic receptors on immune cells. For example, bromocriptine and other DAs have been reported to reduce autoantibody production, alter lymphocyte function, and suppress macrophage IFN-γ production in autoimmune disease models and *in vitro* systems (120, 121). Whether these effects translate to clinically meaningful immunosuppression in patients with DA-treated prolactinomas remains unclear. Consequently, DAs can simultaneously reduce PRL secretion and have been associated with tumor fibrosis, compounding immunosuppressive effects.

Therefore, it is plausible that the fibrotic environment in prolactinomas impedes immune infiltration and contributes to tumor evasion, yet this hypothesis lacks direct evidence. There is currently a paucity of data regarding immune-cell populations, cytokines, checkpoint molecules, and spatial immune exclusion particularly within the context of fibrotic prolactinomas before and after DA therapy. Utilizing DA-resistant prolactinoma organoids, integrated with 3D co-culture systems and spatial transcriptomics (36), is essential for elucidating these mechanisms and identifying novel immunotherapeutic targets to improve clinical outcomes.

### 5.4 The effects of fibrotic change on prolactinoma angiogenesis

Angiogenesis plays a significant role in the TME of fibrotic tumors (122–124). A study by Chen et al. suggests that prolactinomas pre-treated with DAs exhibit increased intra-tumoral fibrosis and DA-pretreated macroadenomas present more intraoperative blood loss (200mL (interquartile range (IQR): 100-400) vs 175mL (IQR: 100-300), P = 0.014) (14). Growing evidence supports that the fibrotic TME actively remodels tumor vasculature through biochemical and biomechanical mechanisms, resulting in both surgical challenges and treatment resistance (122–124). The increase in intraoperative blood loss observed following DA pretreatment may reflect greater mechanical fragility of fibrotic tissue, altered vascularity, or both; however, this finding does not conclusively demonstrate that fibrosis directly induces vascular fragility or leakage. It is noteworthy that this fibrosis-driven vascular remodeling occurs within a broader context of heterogeneous angiogenesis in prolactinomas, where microadenomas are often hypovascular, but invasive macroadenomas are hypervascular (125, 126).

In other tumors, fibrotic change in the TME is considered to drive pathological angiogenesis (21, 29, 127). However, in prolactinomas, direct evidence for a causal link between fibrosis and abnormal vascular architecture remains elusive. Activated TAFs secrete abundant pro-angiogenic factors such as VEGF and TGF-β, directly stimulating endothelial cell proliferation and migration (21). Alterations in VEGF expression may be related to the activation of fibrotic pathways (e.g., ERK1/2 and FAK pathways), potentially through the regulation of ECM production and cell-cell interactions (128, 129). More critically, the hallmark of fibrosis—excessive deposition and cross-linking of the ECM—impacts vascular biology via mechanotransduction. Increased ECM stiffness has been shown to promote vascular growth and disrupt VE-cadherin interactions, leading to enhanced vascular permeability and structural instability (130). Thus, the rigid fibrotic niche may concurrently foster the development of fragile, leaky, and disorganized vessels, and similar mechanisms have been hypothesized to contribute to intraoperative bleeding risk in prolactinomas.

This pro-angiogenic milieu is closely linked to DA resistance. For instance, endothelial cell-specific molecule-1 (ESM1/Endocan) has been identified as a marker of neoangiogenesis in prolactinomas. Its expression and associated microvessel density are significantly higher in DA-resistant tumors compared to sensitive ones (131). Functionally, silencing ESM1 downregulates key angiogenic genes (e.g., vascular endothelial growth factor receptor 2 (VEGFR2), FGF2 and CD34) and inhibits endothelial tube formation, positioning ESM1 as a potential molecular node connecting the fibrotic TME, abnormal angiogenesis, and drug resistance (131). Data regarding ESM1 support an association between angiogenesis and DA resistance, but fail to establish the complete fibrosis—angiogenesis—resistance axis. Furthermore, matrix metalloproteinases (MMPs), involved in ECM remodeling during fibrosis, can cleave matrix components to release growth factors and influence endothelial cell behaviors, creating an additional link between fibrosis and angiogenesis (132–134). While an earlier study suggested bromocriptine treatment did not affect tumor angiogenesis (135), it did not analyze vascular phenotypes within the context of fibrosis. Given that fibrosis is a dynamic process following DA therapy, assessing vasculature independent of fibrosis may yield an incomplete picture. Future studies should employ spatial multi-omics to directly profile vasculature within fibrotic regions and develop non-invasive imaging techniques to assess these vascular abnormalities *in vivo*, ultimately guiding more precise clinical management.

## 6 Future research directions and preclinical strategies to mitigate fibrosis in prolactinomas

Although surgical intervention remains the primary approach for treating DA-resistant prolactinomas, there is an urgent need to explore novel pharmacological therapies for certain invasive or severely fibrotic adenomas. While several anti-fibrotic strategies are promising preclinically, none have been validated in prolactinoma patients. Therefore, the approaches discussed below should be regarded as hypotheses for future research and potential therapeutic avenues rather than current clinical recommendations (**Figure 4**).

**Figure 4:**
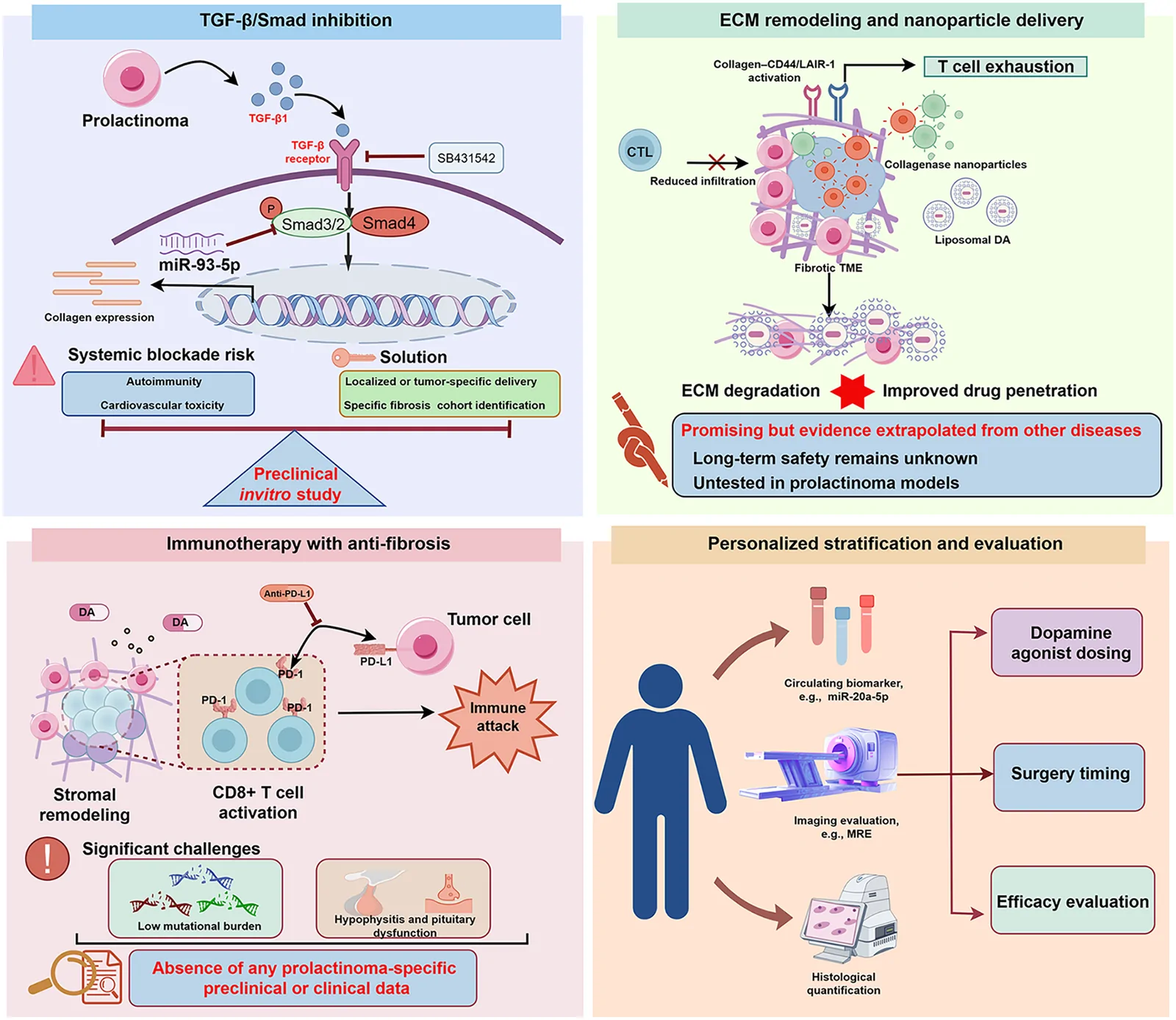
Future research directions and preclinical strategies for mitigating fibrosis in prolactinomas. DA, Dopamine agonist; ECM, Extracellular matrix; CTL, Cytotoxic T lymphocyte.

### 6.1 Targeting the TGF-β/Smad pathway: the most directly supported preclinical approach

Inhibiting the TGF-β signaling, the pathway most consistently implicated in fibrogenesis, has shown efficacy in mitigating fibrosis and modulating the immune landscape in other cancers, and is currently under investigation as a therapeutic strategy to overcome DA-associated fibrosis (64). The TGF-β1/Smad3 signaling-specific inhibitor SB431542 has shown promise in preventing fibrosis and improving drug resistance in prolactinomas (62). *In vitro* studies demonstrated that stimulation with TGF-β1 increased collagen expression in HS27 fibroblasts and diminished the sensitivity to bromocriptine in rat MMQ cells when co-cultured with TGF-β1-treated HS27 cells. SB431542 effectively reversed these effects, suggesting its potential utility as a novel treatment strategy (62). These findings provide proof of concept for the hypothesis that targeting the TGF-β/Smad axis may attenuate fibrosis and partially reverse drug resistance in an *in vitro* model relevant to prolactinoma. Additionally, miR-93-5p has been reported to enhance the drug resistance of prolactinoma cells by regulating TGF-β1/Smad3-dependent fibrosis, inhibiting miR-93-5p could reduce fibrosis and drug resistance (60)(**Figure 4**). These observations suggest that the TGF-β/Smad pathway and its regulatory miRNAs constitute promising targets for future investigation. However, the clinical translation of these anti-fibrotic agents faces significant hurdles. Systemic inhibition of TGF-β1 carries risks of autoimmunity and cardiovascular toxicity (136–138). Therefore, developing localized delivery methods (e.g., via targeted nanoparticles or sustained-release formulations administered during surgery) or identifying prolactinoma-specific fibrotic vulnerabilities will be crucial to maximize efficacy while minimizing off-target effects (139, 140).

### 6.2 ECM-targeting and nanoparticle-based delivery: promising but extrapolated from other diseases

Beyond direct antifibrotic agents, targeting the TME to degrade the ECM barrier has been proposed as a strategy to improve drug permeability and immune response (141–143). The fibrotic stroma not only acts as a physical barrier but also fosters an immunosuppressive environment by impeding CTL infiltration and promoting immune exhaustion through collagen-receptor interactions such as the collagen-CD44 axis and leukocyte associated immunoglobulin like receptor 1 (LAIR-1)-mediated T cell inhibition (144, 145). Modulating these pathways can enhance immune surveillance and boost immunotherapies. Advanced delivery systems are essential to enable therapeutic agents to penetrate effectively. Strategies that disrupt collagen-receptor signaling or degrade ECM components using collagenase-loaded nanoparticles have shown promise in reducing matrix stiffness and enhancing chemotherapeutic efficacy in other fibrotic tumors, such as hepatocellular carcinomas, suggesting translational potential for fibrotic prolactinomas (146). In liver fibrosis, nanoparticles such as inorganic nanoparticles, liposomes, and nanomicelles target hepatic stellate cells to improve therapy (147). The investigation of nanoparticles for the delivery of pharmacological agents or gene therapy in the treatment of prolactinomas merits significant attention. In a mouse model study, liposome-encapsulated bromocriptine demonstrated efficient penetration into the intracranial space, contributing to elevated concentrations of bromocriptine within the hypothalamus and a significant reduction in PRL levels (148).

However, these approaches have not been tested in prolactinoma models, and their applicability to benign neuroendocrine tumors remains uncertain. Pituitary adenomas differ substantially from carcinomas in stromal composition, vascularity, and immune contexture (149). Furthermore, the safety of long-term nanocarrier use remains unresolved. Therefore, ECM-degrading and nanoparticle-based strategies represent future research directions requiring extensive validation, rather than near-term therapeutic options for fibrotic prolactinomas. Whether specifically targeting cells with elevated TGF-β1 or miR-93-5p expression could enhance therapeutic efficacy in fibrotic prolactinomas remains to be investigated (**Figure 4**).

### 6.3 Combining anti-fibrotic strategies with immunotherapy: an untested hypothesis

Once the stromal barrier undergoes remodeling by above-discussed methods, it becomes feasible to combine this process with immune-mediated attacks. This approach is supported by the research showing that functioning pituitary adenomas, particularly somatotroph and lactotroph tumors, exhibit higher PD-L1 expression (23, 150). These findings provide a rationale for the combined use of anti-PD-L1 therapy and DAs. This therapeutic strategy may be further enhanced by the activation of anti-tumor immune cells, such as CD8+ T cells, which have been observed to increase subsequent to cabergoline treatment (18, 23). However, it is imperative to prospectively address several significant challenges, including the typically low mutational burden feature of pituitary adenomas, often referred to as a “cold tumor” phenotype (151, 152), the potential risk of immunotherapy-induced hypophysitis and pituitary dysfunction (153–156), as well as the biocompatibility associated with the long-term deployment of nanocarriers (**Figure 4**). Given the absence of any prolactinoma-specific preclinical or clinical data, combination anti-fibrotic and immunotherapy approaches should be considered speculative and not yet actionable.

The success of these advanced therapies relies on personalized patient categorization and timing. Prospective validation is required before fibrosis assessment can be used to guide DA dosing or determine surgical timing (13, 16). Current evidence does not support altering clinical practice based solely on fibrosis markers. While it is hypothesized that fibrotic prolactinomas may benefit from early surgical resection or adjunctive antifibrotic therapy, this premise warrants prospective evaluation. In addition, regarding molecular biomarkers, circulating miR-20a-5p has been associated with cabergoline responsiveness in prolactinoma patients (33). However, its association with fibrosis has not been directly established, and it cannot yet be regarded as a biomarker for fibrosis risk. Future studies should specifically investigate the correlation between miR-20a-5p (or other circulating markers) and histological fibrosis as well as clinical outcomes in DA-treated prolactinoma patients. This precision medicine approach seeks to balance effective tumor control with reducing fibrosis-induced morbidity, ultimately improving patient prognosis (**Figure 4**).

## 7 Conclusion

DAs are the first-line treatments for prolactinomas, but their association with tumor fibrosis in a subset of patients introduces a significant therapeutic paradox. The evidence synthesized in this review suggests a possible perspective: DA-associated fibrosis may be an active, tumor-adaptive process that contributes to a fibrotic microenvironment with potential implications for therapeutic response within the TME. However, current evidence is largely associative and fails to rule out the competing hypothesis that fibrosis is primarily a passive consequence of tumor involution and tissue remodeling. It has been hypothesized that this fibrotic stroma may contribute to drug resistance, interfere with immune surveillance, and complicate surgical resection by altering tissue architecture. These proposed effects warrant direct experimental validation in prolactinoma-specific models.

There are three main challenges to address as we move forward: (i) deciphering the precise molecular blueprint of this fibrotic niche, including the role of stromal reprogramming, to enhance the effectiveness of drug treatments; (ii) creating standardized clinical methods, including advanced imaging and biomarkers, for non-invasive assessment of fibrosis risk and changes; and (iii) clarifying whether fibrosis assessment can reliably guide the optimal, yet currently uncertain, timing of surgery.

This review has several important limitations that should be acknowledged. First, most clinical evidence is derived from retrospective surgical series with inherent selection bias and variable definitions of fibrosis. Second, histopathological cohorts are generally small and often lack standardized fibrosis scoring systems, limiting comparability across studies. Third, DA regimens are highly heterogeneous in terms of agent, dose, duration, and prior treatment history, which may confound the observed associations. Fourth, preclinical models of prolactinoma fibrosis remain scarce; many mechanistic insights are extrapolated from other tumor types or fibrotic diseases. Fifth, the directionality and causality of the fibrosis–resistance relationship remain unresolved.

Consequently, future research should determine the feasibility of integrating fibrosis assessment, using standardized histopathological, radiological, and biomarker approaches, into clinical decision-making to inform personalized treatment strategies. To date, however, sufficient evidence to support such integration remains lacking. A promising future direction involves combining DA therapy with agents targeting the fibrotic stroma to enhance treatment sensitivity. However, this combinatorial approach remains purely investigational, and whether it improves long-term outcomes remains to be confirmed through rigorous preclinical and clinical validation.
